# Chicago Veterinary College

**Published:** 1901-07

**Authors:** 


### AMONG THE COLLEGES.

### CHICAGO VETERINARY COLLEGE.

THE Chicago Veterinary College has materially altered and extended its curriculum to make room for three additional members to its faculty who have attained enviable reputations as teachers and practitioners in Chicago. They are Drs. L. A. Merillat, Ed. Merillat, and James Wright. Dr. L. A. Merillat takes the subject of principles and practice of surgery; Dr. Ed. Merillat, physiology and demonstrator of anatomy, and Dr. James Wright, cattle, sheep, and swine pathology and obstetrics. These changes will add strength and thoroughness to the very good course heretofore given by this old and reputable institution, and will certainly add materially to its popularity, and will be a cause for increase in the attendance of students. The course in canine and feline pathology was somewhat interfered with last session by the ill health of Dr. C. A. White, but now that he has completely recovered the subject will receive an exhaustive consideration at his hands.

